# Objective and subjective comparison of virtual monoenergetic vs. polychromatic images in patients with pancreatic ductal adenocarcinoma

**DOI:** 10.1007/s00330-019-06116-9

**Published:** 2019-03-19

**Authors:** Lucian Beer, Michael Toepker, Ahmed Ba-Ssalamah, Christian Schestak, Anja Dutschke, Martin Schindl, Alexander Wressnegger, Helmut Ringl, Paul Apfaltrer

**Affiliations:** 1 Department of Biomedical Imaging and Image-guided Therapy, Medical University of Vienna, Waehringer Guertel 18-20, 1090 Vienna, Austria; 2 Department of Surgery, Medical University of Vienna, Vienna, Austria; 3 Institute of Clinical Radiology and Nuclear Medicine, University Medical Center Mannheim, Medical Faculty Mannheim, Heidelberg University, Mannheim, Germany

**Keywords:** Pancreatic neoplasms, Multidetector computed tomography, Signal-to-noise ratio

## Abstract

**Objectives:**

The aim of this study was to assess the objective and subjective image characteristics of monoenergetic images (MEI[+]), using a noise-optimized algorithm at different kiloelectron volts (keV) compared to polyenergetic images (PEI), in patients with pancreatic ductal adenocarcinoma (PDAC).

**Methods:**

This retrospective, institutional review board-approved study included 45 patients (18 male, 27 female; mean age 66 years; range, 42–96 years) with PDAC who had undergone a dual-energy CT (DECT) of the abdomen for staging. One standard polyenergetic image (PEI) and five MEI(+) images in 10-keV intervals, ranging from 40 to 80 keV, were reconstructed. Line-density profile analysis, as well as the contrast-to-noise ratio (CNR) of the tumor, the signal-to-noise ratio (SNR) of the regular pancreas parenchyma and the tumor, and the CNR of the three main peripancreatic vessels, was calculated. For subjective quality assessment, two readers independently assessed the images using a 5-point Likert scale. Reader reliability was evaluated using an intraclass correlation coefficient.

**Results:**

Line-density profile analysis revealed the largest gradient in attenuation between PDAC and regular tissue in MEI(+) at 40 keV. Low-keV MEI(+)reconstructions at 40 and 50 keV increased CNR and SNR compared to PEI (40 keV: CNR 46.8 vs. 7.5; SNR_Pankreas_ 32.5 vs. 15.7; SNR_Lesion_ 13.5 vs. 8.6; *p* < 0.001). MEI(+) at 40 keV and 50 keV were consistently preferred by the observers (*p* < 0.05), showing a high intra-observer 0.937 (0.92–0.95) and inter-observer 0.911 (0.89–0.93) agreement.

**Conclusion:**

MEI(+) reconstructions at 40 keV and 50 keV provide better objective and subjective image quality compared to conventional PEI of DECT in patients with PDAC.

**Key Points:**

*• Low-keV MEI(+) reconstructions at 40 and 50 keV increase tumor-to-pancreas contrast compared to PEI.*

*• Low-keV MEI(+) reconstructions improve objective and subjective image quality parameters compared to PEI.*

*• Dual-energy post-processing might be a valuable tool in the diagnostic workup of patients with PDAC.*

**Electronic supplementary material:**

The online version of this article (10.1007/s00330-019-06116-9) contains supplementary material, which is available to authorized users.

## Introduction

Imaging of patients with pancreatic ductal adenocarcinoma (PDAC) provides preoperative tumor assessment and guidance for either surgery or palliative therapy. However, although routinely performed, preoperative assessment can be challenging, especially for small tumors, due to the low contrast of pancreatic lesions compared to the background pancreatic parenchyma [1].

Contrast-enhanced, thin-section, computed tomography (CT) is the imaging modality of choice to determine resectability [2]. Magnetic resonance imaging (MRI), however, can also be used for local staging [3], but provides additional diagnostic information in the assessment of focal liver lesions [4].

Monoenergetic image (MEI) reconstructions derived from different types of dual-energy CT (DECT) encompassing dual-source [5], split-filter [6], rapid kilovoltage peak (kVp) switching [7], and dual-layer [8] are valuable techniques that improve tissue contrast [9]. DECT has been shown to improve image quality and tissue contrast in diseases of the liver and pancreas [5, 10, 11] and, thus, can increase tumor delineation [12]. However, one drawback of MEI is the increased image noise at low keV levels [13]. Recently, a noise-optimized, virtual monoenergetic reconstruction algorithm has been developed to overcome this limitation (synonym MEI(+)). This technique performs a spatial frequency-based recombination that reduces the image noise of lower energy levels and improves image contrast at higher energies to obtain the best possible image contrast. These novel reconstruction algorithms have been shown to improve signal-to-noise ratio (SNR) and contrast-to-noise ratio (CNR) in oncological and vascular imaging [5, 10, 14, 15].

The aim of this study was to assess the objective and subjective image characteristics of MEI(+) images, using a noise-optimized algorithm at different keV compared to polyenergetic images (PEI), in patients with PDAC.

## Materials and methods

### Patients

This retrospective, single-center, institutional review board-approved data analysis was performed in accordance with the Health Insurance Portability and Accountability Act and the Declaration of Helsinki. The need for written, informed consent was waived due to the retrospective nature of the study. A query of the institution’s radiology information system revealed data sets for 45 patients (18 male, 27 female; mean age, 66 years; range, 42–96 years) with pancreatic cancer who had undergone a clinically indicated, contrast-enhanced, abdominal DE staging CT between 2011 and 2016 for the evaluation of known or suspected pancreatic neoplasm or staging. Inclusion criteria encompassed treatment-naïve patients with both pancreatic parenchymal phase and portal venous phase imaging studies. Exclusion criteria for CT were based on the clinical guidelines of contrast-enhanced CT at our institution: impaired kidney function (GFR < 30 mL/min), severe contrast agent allergy, and inability to give informed consent for the CT examination. Patient demographics, including gender, age, and histopathology of the tumor, were extracted from the hospital’s database.

### DECT image acquisition

Image data were acquired on two different 128-slice, dual-source CT systems (SOMATOM Definition Flash, Siemens Healthineers; SOMATOM Drive, Siemens Healthineers) in dual-energy mode through two x-ray tubes with different kV tube voltages (tube A, 100 kV; tube B, Sn 140 kV), using a tin filter for the high-voltage tube. Automatic exposure control (CAREDose4D, Siemens Healthineers) was used in all scans. Settings for both scanners were as follows: collimation 64 × 0.6 mm; rotation time 0.5 s; pitch 0.9; reference tube current-time product for the 100-kVp tube, 230 mAs; and for the Sn140-kVp tube, 178 mAs. Images were obtained in a craniocaudal direction from the hepatic dome to either the aortic bifurcation or to the symphysis.

A non-ionic contrast agent (Imeron 400, Bracco) with a weight-adapted dose (mean, 107 mL; range, 90–110 mL) was injected at a flow rate of 4 mL/s through a peripheral vein of the forearm or through a central line. All scans were performed in a pancreatic parenchymal phase that was started with a delay of 16 s after a trigger threshold in the abdominal aorta (100 HU) was reached, as well as in a portal venous phase that was acquired 30 s after the end of the arterial phase.

### DECT image reconstruction

Reconstructed CT image data were post-processed on a syngovia workstation (syngo.via, version VB20A; Siemens Healthineers). Standard linear-blended images were reconstructed by applying a blending factor of 0.5 (M_0.5; 50% of the low kV and 50% of the high-kV spectrum). Noise-optimized MEI(+) were reconstructed at 40-, 50-, 60-, 70-, and 80-keV levels while no reconstructions at higher keV levels were performed, as previous studies have suggested optimal keV ranges from 40 to 70 keV [5; 10; 14]. All series were reconstructed as transverse sections with a thickness of 1 mm, an increment of 0.8 mm, and a common soft tissue kernel (D26). These series were then sent to the local PACS.

### Objective image analysis

Line-density analysis, as well as four different quantitative parameters of image quality, was used in the objective image analysis.

### Line-density profile analysis

These reconstructed MEI(+) and linearly blended images were used for the line-density analysis. The tumor was identified on the linearly blended M_0.5 images, and one reader (LB), with 4 years of experience, positioned a line of 10 mm in length and 2 mm in width perpendicular to the tumor margins with one-half the line within the tumor and one-half within the healthy pancreatic tissue. This measurement was applied for each of the five different images per patient at the exact same position, angle, and length. Mean Hounsfield units (HU) were measured within this 10-mm line by the PACS (IMPAX PACS, Agfa HealthCare) software, which was called the line-density profile analysis. The minimum and maximum HU values within these 10 mm were identified. For further statistical work-up, the gradient of the curve was calculated as the difference between the maximum and the minimum HU value within the 10 mm. Line-density analysis was performed in 40 patients, as not enough healthy pancreatic tissue was available in five patients.

All CT images were analyzed on a commercially available PACS workstation by two radiologists with nine (#1 PA) and four (#2 LB) years of experience in abdominal imaging, in consensus, but blinded to both clinical data and reconstruction settings. The maximal tumor diameter was measured in the pancreatic parenchymal phase or portal venous phase, either in the axial or coronal plane in PEI-reconstructed images by reader #2. Attenuation of different areas was measured in Hounsfield units (HU). A region of interest (ROI), as large as possible, was placed in the tumor tissue, avoiding necrotic areas, and in the normal pancreas parenchyma, the paraspinal muscle, and air at the ventral body face. HU and standard deviation (SD) were recorded. For vessel analysis, an ROI covering the maximum lumen was placed in the pancreatic parenchymal phase within the arteria hepatica communis and the arteria mesenterica superior, as well as in the vena mesenterica superior in the portal venous phase.

According to previous studies [10, 14], the formulas with which to determine the quantitative image quality of pancreatic tumors are as follows:

- CNR = HU (pancreas − lesion)/SD (air)
- SNR_pancreas_ = HU (pancreas)/SD (air)
- SNR_lesion_ = HU (lesion)/SD (air)
- CNR_AHP_ = HU (arteria hepatica communis − lesion)/SD (air)
- CNR_AMS_ = HU (arteria mesenterica superior − lesion)/SD (air)
- CNR_VMS_ = HU (vena mesenterica superior − lesion)/SD (air)

### Quantitative assessment of iodine uptake

To quantitatively assess the iodine uptake of the PDAC, an ROI was placed at exactly the same image position in the same reading session as for the objective image evaluation. An ROI placed in the abdominal aorta at the same image position was used for internal normalization of iodine uptake, which was expressed as mg/g tissue. The iodine uptake of the regular parenchyma was compared to that of tumors.

### Subjective image analysis

The same two radiologists independently analyzed all image data. Reader #2 (LB) analyzed images twice, with an interval between the readings of 2 weeks or longer. The assessment of subjective image quality included evaluation of sharpness, image noise, soft tissues, vessel contrast, and overall image quality, and these features were graded on a 5-point Likert scale (5, unacceptable; 4, suboptimal; 3, adequate; 2, good; 1, excellent).

### Statistical analysis

Statistical analysis was performed using SPSS version 22.0 (IBM Cor, 2013) and GraphPad Prism V5. The Kolmogorov-Smirnov test was applied to assess the normality of data distribution. Quantitative variables were expressed as mean ± SD. The paired *t* test was used to compare iodine uptake. Two-way ANOVA with Tukey’s post hoc test was used to evaluate line-density comparisons, as well as SNR and CNR. Intra-observer variability was assessed using an ICC model, with absolute agreements, single measures, and a 95% confidence interval. For inter-observer variability, statistical analyses were calculated for every pairwise combination of observers, using the first evaluation. A two-way random ICC model, with absolute agreement, single measures, and a 95% confidence interval was used. A two-sided *p* value (*p*) less than 0.05 was regarded as statistically significant.

## Results

### Study population

Forty-five patients (18 male, 27 female; mean age, 66 years; range, 42–96 years) with histology-proven PDAC were included in this retrospective study. Of those, 23 patients had undergone surgery, and the remaining 22 patients received systemic therapy without surgery. The maximal tumor diameter measured in the pancreatic parenchymal phase or portal venous phase, either in the axial or coronal plane in PEI-reconstructed images, was 27 mm ± 14 mm (32 mm ± 3 mm vs. 22 mm ± 2 mm in non-operated vs. operated patients, *p* = 0.03).

The mean cumulative CT dose index (CTDI_vol_) of all examinations for the pancreatic parenchymal phase was 13.4 ± 4.6 mGy and 14.1 ± 4.9 mGy for the portal venous phase. Average cumulative DLP was 332.4 ± 132.9 mGy cm for the pancreatic parenchymal phase and 580.8 ± 220.2 mGy cm for the portal venous phase.

### Line-density analysis

**Fig. 1:**
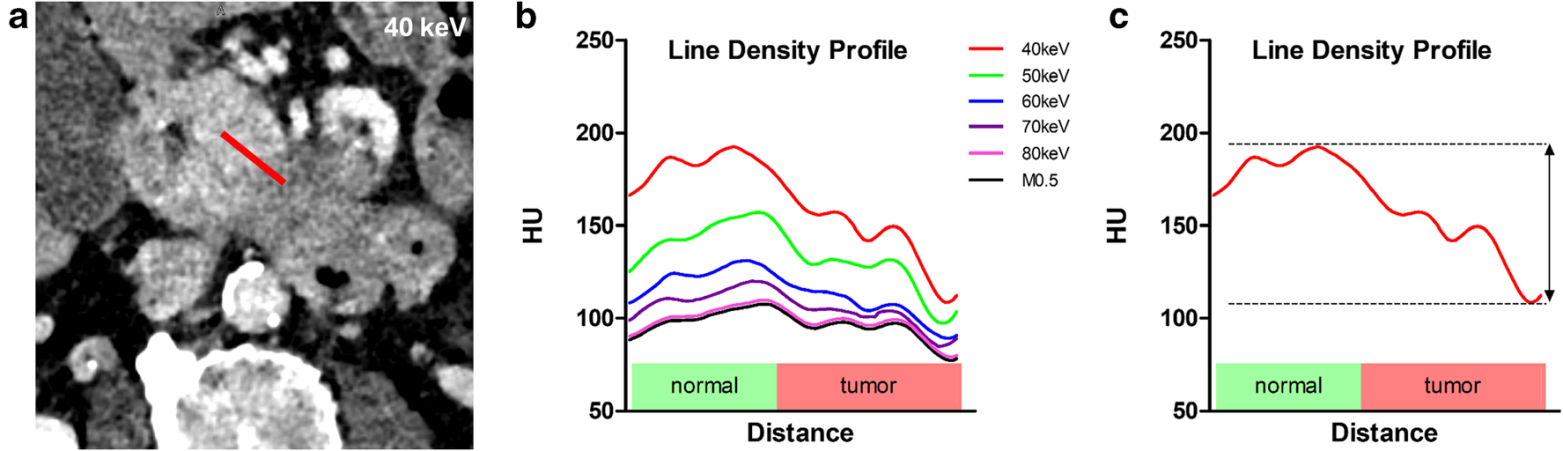
**a** Line-density profile of a 55-year-old male patient with adenocarcinoma of the pancreas, using MEI(+) 40-keV reconstructions. The line was placed perpendicular to the border of the tumor within 1-mm-thick images at the same position in all images evaluated. The line had a length of 10 mm. The minimum and maximum attenuations were used for further calculation. **b** Plot of the line-density profile of the same patient for different image reconstructions. **c** Schematic example of the gradient of those distances that was used for the line-density profile analysis

Tumor line–density analysis revealed the highest contrast differences between tumor and regular pancreatic tissue for the 40-keV MEI(+) reconstructions, followed by the 50-keV, 60-keV, 70-keV, and 80-keV MEI(+) reconstructions and PEI reconstructions for both the pancreatic parenchymal phase and portal venous phase (Fig. 1). The mean difference between the maximum and minimum attenuation within the tumor border, resembling the gradient of the line-density profile, was greatest for the 40-keV images and lowest for the PEI, as shown in Table 1. Except for venous 70-keV and 80-keV MEI(+) reconstructions, the differences were statistically significant between PEI and MEI(+), as well as between different MEI(+) reconstructions (corrected *p* < 0.05).

**Table 1:** Differences between line-density profiles within the tumor border

Images	Mean ± SD	Maximum	Minimum
Pancreatic parenchymal phase			
M_0.5	121 ± 38	251	− 48
40 keV	376 ± 126	776	− 68
50 keV	269 ± 77	400	− 54
60 keV	193 ± 52	373	− 50
70 keV	151 ± 41	316	− 45
80 keV	131 ± 38	261	− 44
Portal venous phase			
M_0.5	101 ± 39	194	− 26
40 keV	310 ± 136	622	− 79
50 keV	231 ± 105	480	− 55
60 keV	155 ± 73	304	− 40
70 keV	126 ± 52	233	− 35
80 keV	109 ± 46	198	− 29

The difference was defined as the maximum Hounsfield units (HU) − minimum HU. All values are given in HU

### Objective image quality parameters—pancreas

**Fig. 2:**
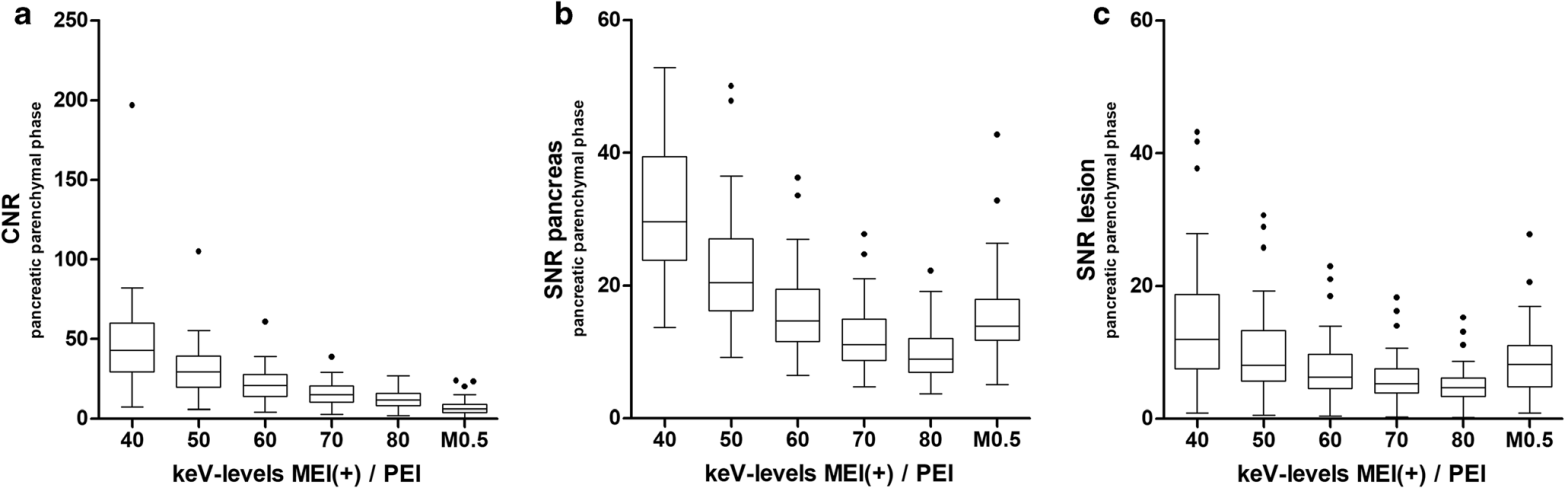
Objective image characteristics for MEI(+) data sets from five different monoenergetic kiloelectron levels ranging from 40 to 80 keV and 0.5-average-weighted PEI (M_0.5). Panel (**a**) contrast-to-noise ratio (CNR) of the pancreas parenchyma at the pancreatic parenchymal phases. Panel (**b**) signal-to-noise ratio (SNR) of the pancreas parenchyma. Panel (**c**) SNR of tumor tissue. Data are given in boxplots, where the whiskers represent a 1.5 IQR. Outliers are given as dots. *n* = 45; *MEI*, monoenergetic images; *PEI*, polyenergetic images

Applying Tukey’s post hoc test, MEI(+) at 40 keV provided the highest CNR of PDCA in the pancreatic parenchymal phase (Fig. 2a), as well as in the portal venous phase (supplementary Fig. 1A). The CNR of 40-keV MEI(+) was significantly higher than the CNR of 50-keV MEI(+), and the CNR of 50-keV MEI (+) was significantly higher than the CNR of 60-keV MEI(+). Except for 80 keV, all MEI (+) had a significantly higher CNR compared to PEI in both phases.

SNR of the pancreas (Fig. 2b) and the tumor (Fig. 2c) in the pancreatic parenchymal phase showed similar results, with the highest values for MEI(+) at 40 keV, followed by 50 keV. In contrast to the CNR, the SNR of MEI(+) at 60 keV was comparable to that of PEI. MEI(+) at 70-keV and 80-keV reconstructions had significantly lower SNR values compared to PEI. The results of the portal venous phase are shown in supplementary Fig. 1B and 1C.

### Objective image parameters—vessels

**Fig. 3:**
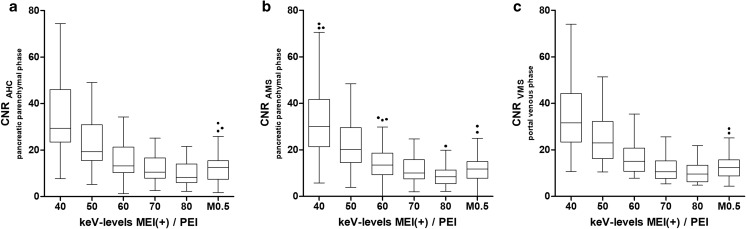
Objective image characteristics for MEI(+) data sets from five different monoenergetic kiloelectron levels ranging from 40 to 80 keV and 0.5-average-weighted PEI (M_0.5). Contrast-to-noise ratio of the **a** arteria hepatica communis (AHC), **b** the arteria mesenterica superior (AMS), and **c** the vena mesenterica superior (VMS). Data are given in boxplots, where the whiskers represent a 1.5 IQR. Outliers are given as dots. *n* = 45; *MEI*, monoenergetic images; *PEI*, polyenergetic images

MEI(+) reconstructions at 40 keV and 50 keV significantly improved the CNR of the arteria hepatica communis (AHC) (Fig. 3a), the arteria mesenterica superior (AMS) (Fig. 3b), and the vena mesenterica superior (VMS) (Fig. 3c) compared to the other MEI(+) (*p* < 0.001) and PEI (*p* < 0.0001) reconstructions. The 40-keV MEI(+) for all vessels analyzed had a significantly higher CNR compared to the 50-keV MEI(+), both of which were higher compared to 60-keV MEI(+). The CNR of all three vessels were not significantly different between MEI(+) at 60 keV, 70 keV, 80 keV, and PEI.

### Iodine uptake

**Fig. 4:**
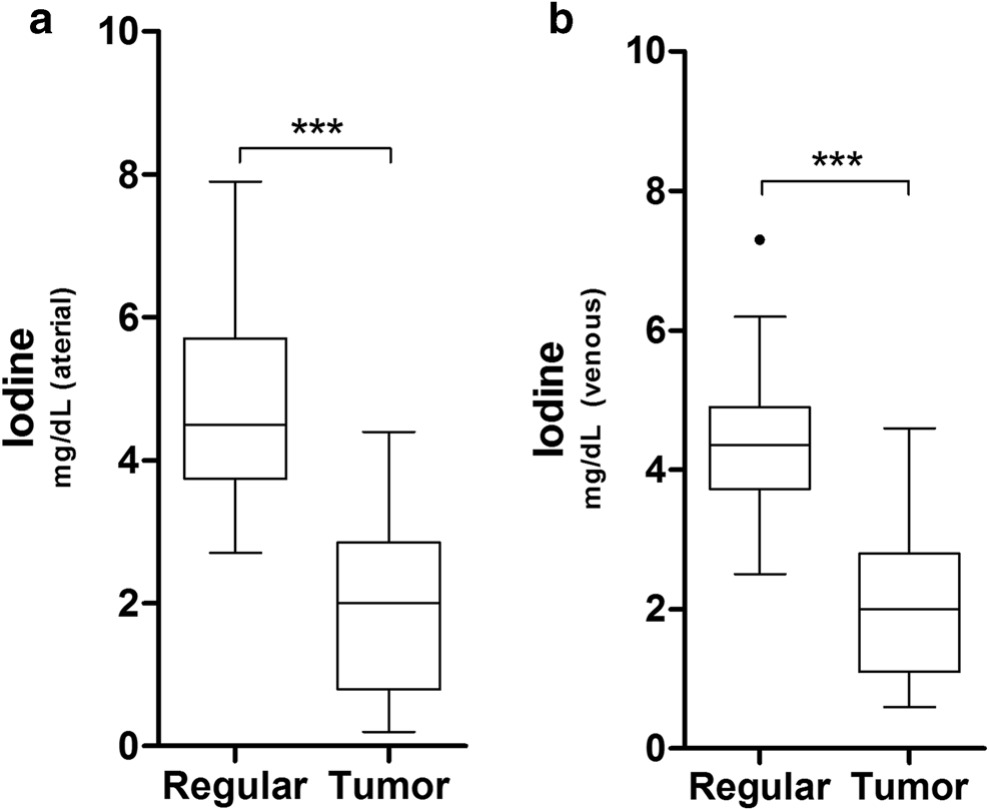
Iodine uptake (mg/dL) was quantified and compared between the regular pancreas parenchyma and tumor tissue. In panel (**a**), the pancreatic phase (arterial) is shown, whereas in panel (**b**), the pancreatic venous phase is depicted. Data are given in boxplots, where the whiskers represent a 1.5 IQR. Outliers are given as dots. *n* = 45

Neoplastic tissue had a significantly reduced iodine uptake compared to regular pancreas tissue in the pancreatic parenchymal phase (2.0 ± 1.1 vs. 4.8 ± 1.3 mg/dL; *p* < 0.0001, Fig. 4a) and in the portal venous phase (2.1 ± 1.1 vs. 4.4 ± 1.0 mg/dL, *p* < 0.0001, Fig. 4b).

### Subjective image quality

**Fig. 5:**
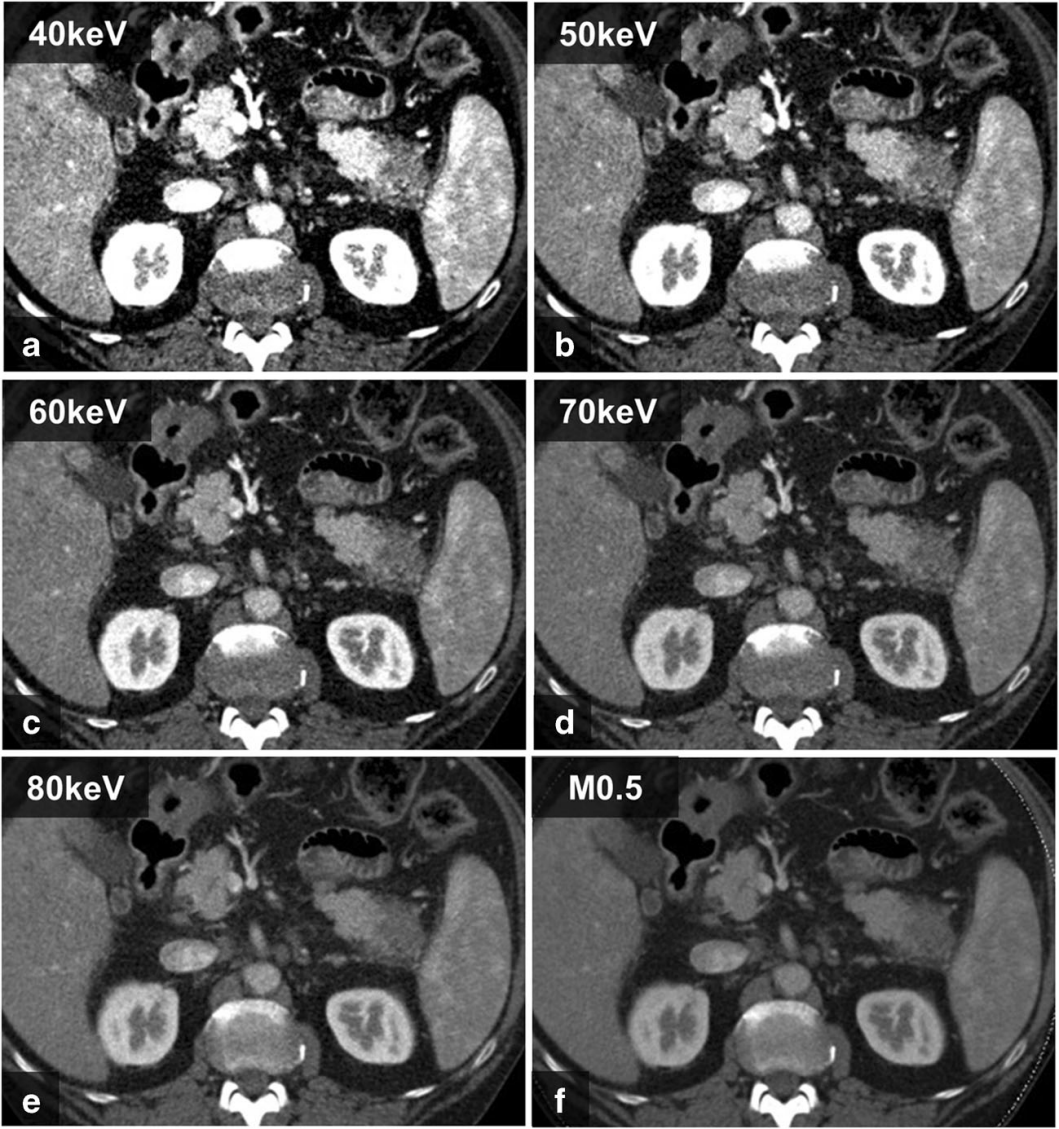
Transversal, axial images of a 54-year-old male patient with pancreatic adenocarcinoma of the pancreatic tail. The contrast-to-noise ratio and the signal-to-noise ratio were highest on the MEI(+) with 40 keV (**a**), followed by 50 keV (**b**), 60 keV(**c**), and 70 keV (**d**). There were no differences on MEI(+) at 80 keV (**e**) compared to average-weighted PEI M_0.5 (**f**). Subjective image quality was rated best at 40 keV and 50 keV. *MEI*, monoenergetic image; *PEI*, polyenergetic images

**Fig. 6:**
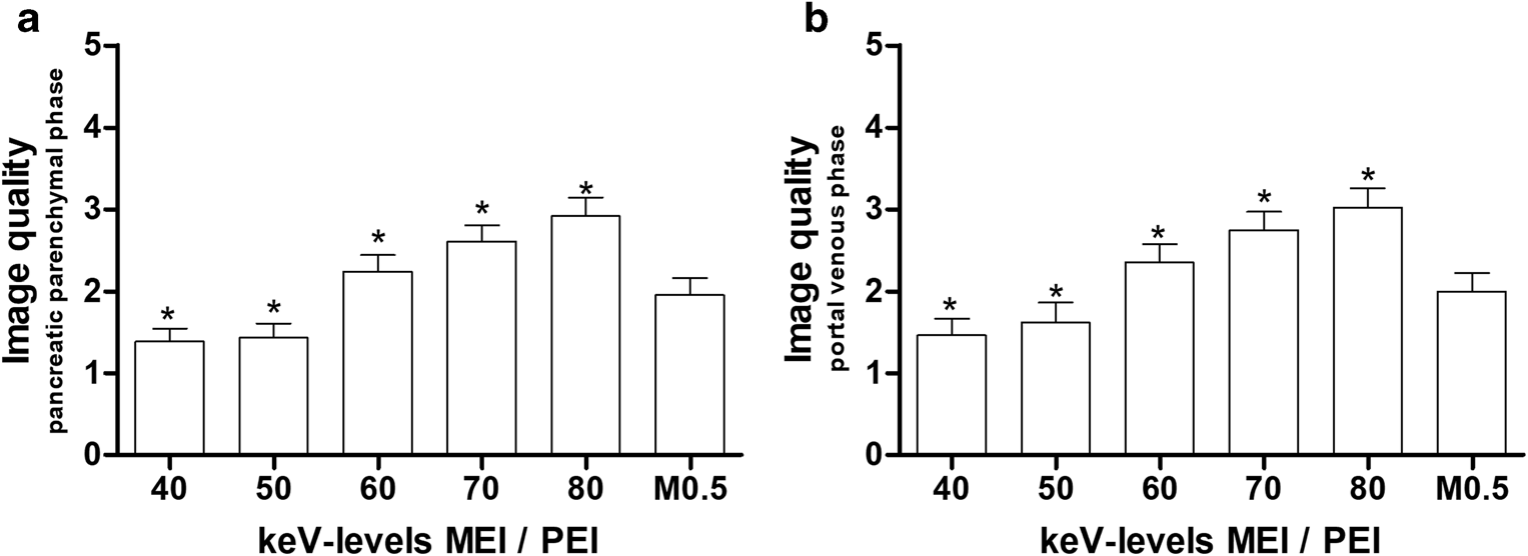
Subjective image characteristics for MEI(+) datasets from five different monoenergetic kiloelectron levels ranging from 40 to 80 keV and 0.5-average-weighted PEI (M_0.5) at the pancreatic parenchymal phase (**a**) and the portal venous phase (**b**). A 5-point scale ranging from perfect image quality (1) to inappropriate image quality (5) was used for quantification. Data are given as bars with the mean + 95% CI. *n* = 45; *MEI*, monoenergetic image; *PEI*, polyenergetic images

Subjective image quality was assessed in 40–80-keV MEI(+) and PEI reconstructions for the pancreatic parenchymal phase (Fig. 5) and for the portal venous phase (Fig. 6a, b). The 40-keV and 50-keV images were rated significantly better than all other MEIs, as well as all PEIs, both in the arterial phase and in the pancreatic venous phase (*p* < 0.001). The subjective image quality of MEIs+ at 60–80 keV was lower than that of PEI (*p* < 0.001). There were no differences in image quality between MEIs+ at 40 keV and 50 keV (*p* > 0.05). The ICCs for intra- and inter-observer variability in terms of image quality were 0.937 (0.92–0.95) and 0.911 (0.884–0.925) for the pancreatic parenchymal phase and 0.918 (0.895–0.935) and 0.915 (0.891–0.933) for the portal venous phase, respectively.

## Discussion

In this study, we evaluated the impact of MEI(+) reconstructions on objective and subjective image parameters of malignant tissue and peripancreatic vessels in patients with PDAC. This algorithm demonstrated significantly better objective and subjective image parameters at lower keV (40–50) compared to PEI and MEI(+) images at higher keV (60–80). Thus, the results indicate that 40–50 keV MEI(+) reconstructions should be the preferred reconstruction technique for the diagnostic workup of patients with PDAC, if a DECT scanner is available.

Several studies in the field of oncological and vascular imaging have shown that MEI(+) reconstructions are superior to MEI and PEI, providing excellent image quality due to significantly lower image noise at similar attenuation levels [5, 8–10, 15–18]. Only two of these studies investigated image quality in patients with PDAC [10, 17]. These studies evaluated MEI(+) images obtained in the pancreatic parenchymal phase in a final set of 30 [10] and three [17] PDAC patients, respectively. Both studies favored MEI(+) reconstructions in terms of subjective and objective image parameters. In this study, we confirmed, in large part, their observations and have added more evidence using a much larger patient cohort; a dual-energy scanning technique for both the pancreatic parenchymal phase, as well as the portal venous phase; a line-density profile analysis, a visualization technique for the contrast at the tissue/tumor border; and a variety of additional parameters, such as image quality of the peripancreatic vessels. We found that the best CNR and SNR for the assessment of the PDACs were obtained with MEI(+) at 40–50 keV for both contrast phases.

By performing a line-density profile analysis, we assessed an additional parameter of objective image quality and found that MEI(+) reconstructions increase the contrast at the border zone between malignant and regular pancreatic tissue. Line-density profile analysis visualizes the contrast gradient at the pancreatic tissue-to-tumor interface and enables easy comparison of different reconstruction algorithms [12]. The gradient of the contrast curve has been calculated from the maximum and minimum HU values at the pancreatic tissue/tumor border as a marker for tumor delineation. Tumor delineation was highest on the MEI(+) images at 40 keV followed by the 50–70-keV MEI(+) and PEI reconstructions, which is in-line with the other objective image parameters.

To further extend the analysis, we performed an objective evaluation of peripancreatic vessels, as their preoperative assessment is crucial for treatment decision-making. Importantly, MEI(+) at low keV levels significantly increased the CNR, which peaked at 40 keV MEI(+) reconstructions. This further indicates that MEI(+) reconstructions might help not only to identify and delineate PDAC but also to support the assessment of tumor spread along the peripancreatic vessels.

The objective image noise characteristics of MEI(+) reconstructions observed in this study are comparable with recent investigations that show increased noise for lower energy levels [5, 10, 17]. Interestingly, while subjective image parameters were best for MEI(+) reconstructions at 55 keV in the study by Frellesen, in our study, both readers favored the 40-keV and 50-keV MEI(+). A potential explanation could be drawn from differences in slice thickness (1 mm vs. 3 mm). However, as a common denominator, all studies consistently favored the reconstruction technique MEI(+) at lower keVs. We conclude that, although image noise is slightly higher in low MEI(+) keV reconstructions, the net benefit of increased tissue contrast seems to outweigh this drawback. When considering the incorporation of MEI(+) images into the routine workflow, we suggest establishing an initial adoption period in which readers become familiar with the altered signal-to-noise ratio of the reconstructed images.

Besides the reconstruction of MEI(+), DECT allows the calculation of material-specific iodine images. Quantification of iodine concentrations has been shown to provide beneficial information regarding tumor delineation and detection. In patients with hypervascular liver tumors, material-density iodine images served as objective markers of image quality and also provided a slight increase in the diagnostic confidence for the differentiation between benign and malignant tissue [19]. Moreover, in patients with hepatocellular carcinoma, iodine uptake showed a high correlation with arterial tumor perfusion [20], indicating that iodine concentration can be used as a surrogate parameter for tumor vascularity. In the current investigation, iodine concentrations were significantly lower in tumor tissue compared to normal pancreas, which is in-line with previous studies [21].

Iodine images can further be used to assess the post-surgical, local recurrence of pancreatic cancer. According to Parakh et al, material-specific iodine images improved radiologists’ confidence for both detecting and excluding the local recurrence of pancreatic cancer [22]. Based on our experience, we believe that material-specific iodine images, in combination with low-keV MEI(+), might provide additional valuable information, especially for the detection of small, hypoattenuating tumors. However, improved tissue contrast can potentially also lead to false-positives, and therefore, this question should be addressed by a sub-group analysis in larger patient cohorts or in prospective multi-center studies.

Our study does suffer from several limitations. Despite the advantages of DECT with MEI(+) on objective and subjective image parameters, there are no verified reports about an improvement in the tumor detection rate, as yet [23]. However, our study was not focused on the detection rate but, rather, on the optimal protocol to be used for the detection of tumor borders. Based on our experience, MEI(+) with low keV mainly improves reader confidence for pancreatic lesions. Indeed, this impression might be biased by the retrospective study design, which included only patients with proven pancreatic cancer. A further limitation that has to be mentioned is that image analysis was based on standard linearly blended M_0.5 images and MEI(+) reconstructions. Other blending factors, such as M_0.3 or M_0.4, as well as 120-kV single-energy CT, were not assessed since previous studies have shown only minor differences in lesion attenuation between blending factors [24] and single-energy CT data were not available in this patient cohort. We did not evaluate the potential impact of MEI(+) reconstructions on the image quality and detectability of liver and lymph-node metastases. Finally, because we included not just surgical patients, this study lacks pathological and surgical correlation, and those data would have been valuable for further analysis. These tasks can serve as the basis for further research projects that could assess the value of MEI(+) reconstructions in abdominal imaging.

In conclusion, this study showed that, in patients with PDAC, MEI(+) reconstructions at low keV levels, using a noise-optimized algorithm as part of a dual-source DECT protocol, provide improved objective and subjective image quality compared to PEI. Consequently, MEI(+) reconstructions might be used to improve the diagnostic image quality of CT for the assessment of PDAC and peripancreatic vessels.

## Electronic supplementary material

ESM 1 (DOCX 2585 kb)

## Abbreviations

CNR: Contrast-to-noise ratio
DECT: Dual-energy computed tomography
HU: Hounsfield units
MEI: Monoenergetic image
PDAC: Pancreatic ductal adenocarcinoma
PEI: Polyenergetic images
ROI: Region of interest
SNR: Signal-to-noise ratio
