# Supplementary material for: Objective and subjective comparison of virtual monoenergetic vs. polychromatic images in patients with pancreatic ductal adenocarcinoma
Source: Eur Radiol. 2019 Mar 19;29(7):3617–25. doi: 10.1007/s00330-019-06116-9 (PMC6554239; doi:10.1007/s00330-019-06116-9)
Supplement: Supplementary file 1 — (DOCX 2585 kb) [file 330_2019_6116_MOESM1_ESM.docx]

**Objective and Subjective Comparison of Virtual Monoenergetic vs. Polychromatic Images in Patients with Pancreatic Ductal Adenocarcinoma**

Beer et al.


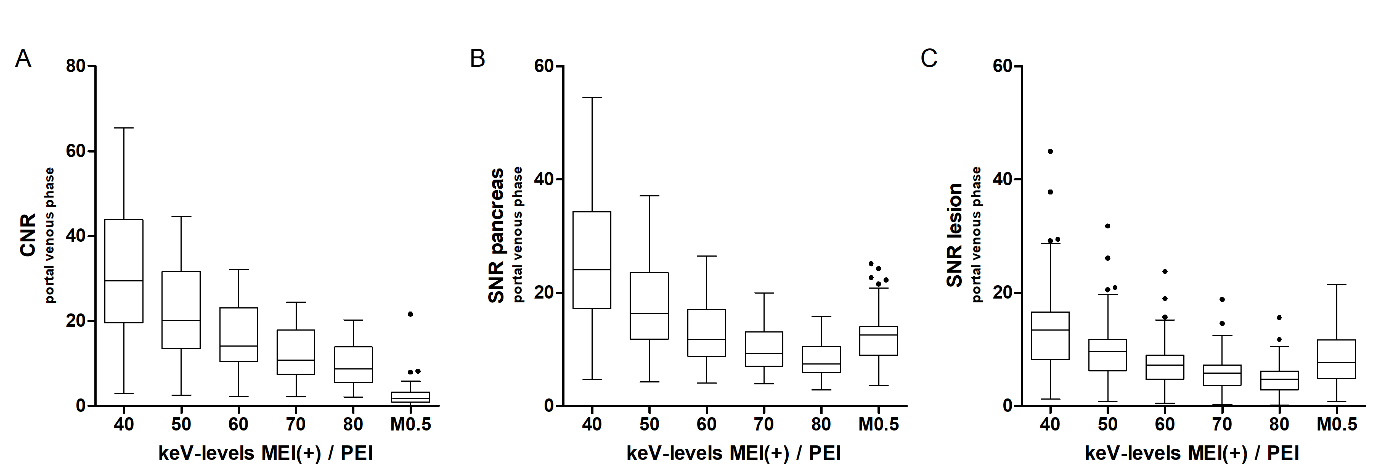


| **sFigure 1.**  Objective image characteristics for MEI(+) data sets from five different monoenergetic kiloelectron levels ranging from 40 keV to 80 keV and 0.5-average-weighted PEI (M_0.5). Panel (A) contrast-to-noise ratio (CNR) of the pancreas parenchyma at the portal venous phases. Panel (B) signal-to-noise ratio (SNR) of the pancreas parenchyma. Panel (C) SNR of tumor tissue. Data are given in boxplots, where the whiskers represent a 1.5 IQR. Outliers are given as dots. n=45; *MEI, monoenergetic images; PEI, polyenergetic images.* |
| --- |
